# Socio-cultural influences on children’s feeding habits and feeding frequencies in Ouagadougou, Burkina Faso: a retrospective survey

**DOI:** 10.1186/s40795-023-00698-w

**Published:** 2023-03-09

**Authors:** Sanogo Bougma, François Tapsoba, Judith Nomwendé Semporé, Sibiri Bougma, Prudence Dounia, Laurencia Toulsoumdé Songré-Ouattara, Aly Savadogo

**Affiliations:** 1 Laboratory of Applied Biochemistry and Immunology (LaBIA)/Department of Biochemistry and Microbiology, University Joseph KI-ZERBO, Ouagadougou, 03 PO Box 7021, Burkina Faso; 2 Laboratory of Biochemistry, Biotechnology, Food Technology and Nutrition (LABIOTAN)/Department of Biochemistry and Microbiology, University Joseph KI-ZERBO, Ouagadougou, 09 PO Box 848, Burkina Faso; 3 Department of Food Technology (DTA)/Research Institute of Applied Sciences and Technology (IRSAT), Ouagadougou, 03 PO Box 7047, Burkina Faso

**Keywords:** Meal frequency, Breastfeeding mode, Family meals, Social status, Ouagadougou

## Abstract

**Background:**

From 6 months of age, children need, in addition to breast milk, a complementary food whose nutritional composition meets their needs. However, low consumption of child-specific foods in favor of adult foods has been documented. Thus, the lack of adaptation of children to family feeding conditions has been the source of frequent malnutrition in some low-income countries. In Burkina Faso, little data is available on family-type food consumption by children. The objective was to describe the socio-cultural influences on feeding habits and food consumption frequencies of infants aged 6–23 months in Ouagadougou.

**Methods:**

The study was conducted from March to June 2022 using a structured questionnaire. A reminder of the previous 24 h’ meals was used to assess 618 children's food consumption. Mother–child pairs were selected using the simple random sampling method, and data collection was done by the interview method. Sphinx V5, IBM SPSS Statistics 20.0 and XLSTAT 2016 software were used to process data.

**Results:**

Influences between the consumption of certain foods and the mother’s social status were observed. The most consumed foods are simple porridges (67.48%), Tô/rice (65.70%), cookies and cakes (62.94%), juices and sweetened drinks (62.94%). Cowpeas (17.31%), improved porridge (13.92%) and eggs (6.63%) are the least consumed. The most meals frequency was three meals daily (33.98%), and children with the minimum daily meal frequency were 86.41%. Principal component analysis showed that the mother's social status influenced the consumption of imported infant flours, fish soups, fruits, juices and sweetened drinks, cookies and cakes, simple porridge, and tô/rice. Concerning the consumption of local infant porridges, 55.72% of the children who consumed them appreciated positively. However, for 57.75% of the parents, the lack of information limits the consumption rate of this type of flour.

**Conclusion:**

High consumption of family-type meals was observed and was influenced by parental social status. In addition, the rate of acceptable meal frequencies was generally high.

## Background

The nutritional situation in Burkina Faso, as in other Sahelian countries, is cause for concern, particularly for young children [1]. The immediate causes of this situation remain insufficient food intake. The fundamental factors of this insufficiency are the low levels of education, high population growth, the general poverty of the population, and particularly of women [2]. A low and non-diversified diet leads to malnutrition [3, 4] and compromises children’s good physical and intellectual growth [5]. Some studies have shown that malnutrition is most often caused by the lack of adaptation of specific individuals or groups of individuals to family food conditions [6]. Burkina Faso is composed of several different ethnic groups, languages, customs, and religions that live side by side daily and engage in everyday activities [7]. This differences results in a multitude of dietary practices and habits which can influence children food consumption.

Reflections on the sociocultural link of food have been developed by some authors [8, 9] to understand the spatial and temporal differences in child malnutrition. Dietary learning starts from the beginning of diversification with, in addition to the consumption of child-specific meals, the consumption of certain family foods [10]. According to some authors, early consumption of family foods would prevent specific food allergies that some children might develop [11]. Learning goes through many of stages, allowing the exploration of new textures, new tastes, and the application of the appropriate gestures to eat. Thus, for some authors, the learning process is a standard set of transmission of symbols and values that allow the child to integrate into the social group to which it belongs [12]. The child is, therefore, regularly brought into contact with various foods and thus with a wide range of tastes. The child thus becomes familiar with the characteristic flavors of family food preparations throughout its development. These environmental influences on infant feeding have led some authors to research and conclude that a child's food preferences are influenced by genetics, by the environment in which it grows up and by the context in which it discovers food [13, 14]. Other studies have also shown that the people around the child also play a considerable role in dietary learning [15].

In Burkina Faso, very few studies have focused on consuming foods not explicitly reserved for children in households. That is why this study was initiated to describe the socio-cultural influences on eating habits and food consumption frequencies of children aged 6 to 23 months in the city of Ouagadougou to get a picture of the situation at the national level on the issue of infant feeding in households.

## Materials and methods

### Survey area

**Fig. 1:**
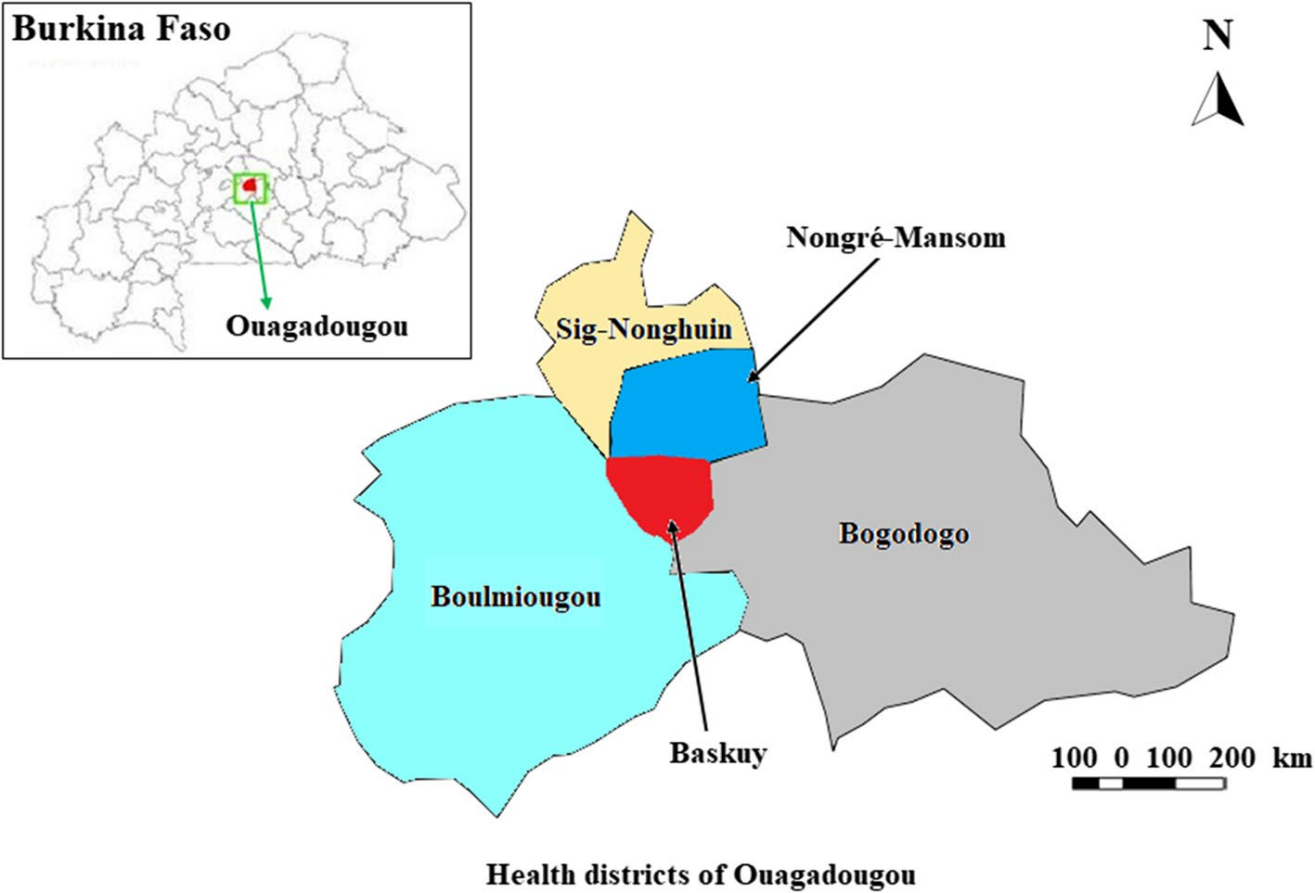
Health map of the city of Ouagadougou. Source: [17]

The study was conducted in the Maternal and Child Health Services (MCHS) of the various health Centers in Ouagadougou from March to June 2022. In terms of health, the city is divided into five (5) health districts with different coverage sizes, all taken into account for the survey (Fig. 1). The choice of Ouagadougou city was justified by the fact that it brings together almost all the social groups of the different regions of the country, which makes it possible to obtain a picture of regional practices. In 2020, the child population of the city of Ouagadougou was estimated at 188,185 children aged 0–24 months [16]. The urban municipality of Ouagadougou is located in the province of Kadiogo in the Center region of Burkina Faso.

### Sample size determination

The sample size was obtained from the following formula (For a population greater than 10,000) [18]. The accepted error rate was 5%, and the confidence coefficient was 1.96, with 95% as a confidence interval. The prevalence rate used in this study was 50%. According to the sample size determination method, 436 mother-infant pairs were planned for this survey. However, the population’s high acceptance of the survey led us to survey more than 640 mother–child pairs. Thus, after sorting and eliminating incorrect responses, poorly filled-out forms, and wrong answers, data from 618 mother–child pairs were retained and analyzed. The collection rate was 146.79%, with an adjusted response rate of 96.56%.

$$\begin{array}{c}\mathbf n=\frac{\mathbf t^{\mathbf2}\mathbf p\left(\mathbf1-\mathbf p\right)}{\mathbf m^{\mathbf2}}\\\begin{array}{cc}\mathrm n=\mathrm{Sample}\;\mathrm{size}\;(\mathbf n=\mathbf{436})&\mathrm t=\mathrm{Confidence}\;\mathrm{coefficient}(\mathrm t=1,96)\;\mathrm{for}\;\mathrm{IC}\;\;95\%\end{array}\\\begin{array}{c}\begin{array}{cc}\mathrm m=\mathrm{Consensus}\;\mathrm{error}\;\mathrm{rate}\;(\mathrm m=5\%)&\mathrm p=\mathrm{Expected}\;\mathrm{prevalence}\;(\mathrm p=50\%)\end{array}\\\mathrm N=\mathrm{childpopulation}\;(\mathbf N\boldsymbol\approx\mathbf{188}\boldsymbol,\mathbf{185}\;\mathbf c\mathbf h\mathbf i\mathbf l\mathbf d\mathbf r\mathbf e\mathbf n\boldsymbol\;\mathbf a\mathbf g\mathbf e\mathbf d\boldsymbol\;\mathbf0\boldsymbol-\mathbf{24}\boldsymbol\;\mathbf{months},\;\mathrm{base}:\;\mathrm{INSD}\;2022,\;5\mathrm{th}\;\mathrm{RGPH}\;\mathrm{locality}\;\mathrm{file})\end{array}\end{array}$$

### Participant sampling methods

The study was a cross-sectional survey using the simple random sampling method. The mother–child pairs were selected randomly after voluntary acceptance of the participant in the five health districts with the results of the national statistical yearbook of health as the sampling frame [19]. The choice to survey all five health clusters was made to cover all social strata and wide geographic distribution.

### Data collection method

The primary data collection method was face-to-face interviews, using a structured questionnaire based on the WHO indicators for assessing infant and young child feeding practices [20]. The general information collected was the child's and mother's name and age, the mother's marital status, education level and occupation and the child's gender. The reminder of meals consumed in the past 24 h was obtained by maternal self-report. The children's mothers were asked to list all the meals consumed by the child and those consumed by the family the previous day before the survey. All the ingredients used in preparing the meals were also listed.

### Data quality control

Firstly, before every interview, the respondents were made aware of the importance of their answers. The questionnaire was also designed to verify past responses with the following questions. In addition, interviewers were trained to detect incorrect answers by returning to some of the answers previously given by respondents and repeating some questions. This technique resulted in satisfactory responses from the mothers. However, after data collection, the forms were sorted to eliminate incorrect responses, poorly filled-out forms, and wrong answers.

### Target population and inclusion criteria

The population consisted of mother–child pairs. The inclusion criteria for the children were age (ranging from 6 to 23 months), state of health (being in good health), attendance at a Health Center in the study area by the mother, and voluntary acceptance of the mother through an informed consent form.

### Analysis of the data

The survey forms were first sorted to eliminate poorly completed forms and those with inconsistent data. Suitable quality forms were entered into Sphinx V5 software and then transferred to IBM SPSS statistics 20.0 software for second sorting before generating numbers and frequencies. Graphs were made on Microsoft Excel 2016 software. XLSTAT 2016 software was used to perform principal component analysis and generate *P*-values using Student's t-test. The threshold of statistical significance was set at *p* < 0.05.

## Results

### Socio-demographic characteristics of the mothers and children

The results of the selection of mother–child pairs are presented in Table 1. Regarding the children's characteristics, most were between 9 and 23 months, with a balance between male and female. Regarding the mothers’ characteristics, the majority were between 26 and 34 years and most had a secondary level. A large majority of the mothers were also married. As for mothers’ occupations, unemployed housewives were the most numerous.

**Table 1:** Socio-demographic characteristics of the participants

Characteristics		Frequencies (%)
**Children’s age groups (*N* = 618)**	**6–8 months**	36.73
	**9–23 months**	63.27
**Children’s gender (*N* = 618)**	**Male**	50.97
	**Female**	49.03
**Mother’s age groups (*N* = 618)**	**17–25 years**	36.41
	**26–34 years**	46.28
	**35–44 years**	17.31
**Education level (*N* = 618)**	**NS**	20.55
	**PL**	23.95
	**SL**	40.78
	**HL**	14.72
**Marital status (*N* = 618)**	**Sin**	4.85
	**Mar**	95.15
**Occupation (*N* = 618)**	**Hw**	43.53
	**Sal**	21.68
	**IS**	34.79

*Legend*: *NS* Note schooled, *PL* Primary level, *SL* Secondary level, *HL* High level, *Sin* Single, *Mar* Married, *Hw* Housewife, *Sal* Salaried, *IS* Informal sector, *N* Number

### Consumption of different types of food according to the feeding method

The results in Table 2 concern the consumption of different foods given to children by their mothers during food diversification. The consumption of fifteen types of food frequently given to children and family meals was evaluated. The results showed that the children consumed simple porridges most, followed by tô/rice. Cookies, cakes, juices, sweet beverages, and fruits were also heavily consumed. The least consumed foods were vegetables, meat, tubers purees, cowpeas and peas, improved porridge and eggs. The comparison between the consumption of different foods according to the mode of feeding shows significant differences concerning the consumption of local infant flours, improved porridges, tô/rice, tubers purees and fruits. By making groupings based on the food groups proposed by the WHO, cereals were predominant in the children's diet, with the consumption of family meals (tô/rice), cookies and porridges. Meat products (fish and meat soups), fruits and sweet juices were also consumed to a large extent. Dairy products, vegetables, legumes (cowpeas and peas), eggs and tubers purees were consumed to a small degree.

**Table 2:** Consumption of food types according to the type of breastfeeding

Type of food	Totals (N)%	Breastfeed (*N* = 590)	Non-breastfeed (*N* = 28)	*P*-value
Industrial milk	(206) 33.33	(178) 30.17	(28) 100.00	0.313
Imported infant flours	(280) 45.31	(274) 46.44	(6) 21.43	0.225
Local infant flours	(193) 31.23	(183) 31.02	(10) 35.71	0.045*
Simple porridges	(417) 67.48	(409) 69.32	(8) 28.57	0.251
Improved porridges	(86) 13.92	(82) 13.90	(4) 14.29	0.009*
Cookies and cakes	(389) 62.94	(380) 64.41	(9) 32.14	0.205
Fish soups	(254) 41.10	(240) 40.68	(14) 50.00	0.065
Meat soups	(142) 22.98	(137) 23.22	(5) 17.86	0.083
Eggs	(41) 6.63	(36) 6.10	(5) 17.86	0.710
Cowpeas and peas	(107) 17.31	(94) 15.93	(13) 46.43	0.290
Tô/rice	(406) 65.70	(387) 62.62	(19) 67.86	0.026*
vegetables	(158) 25.57	(140) 23.73	(18) 64.29	0.275
Tubers purees	(117) 18.93	(111) 18.81	(6) 21.43	0.041*
Fruits	(325) 52.59	(308) 52.20	(17) 60.71	0.048*
Juices and sweet drinks	(389) 62.94	(381) 64.58	(8) 28.57	0.235

*Legend*: *N* Number, *%* Frequency, * Significant level

### Consumption of different types of food according to the mother's social status

**Fig. 2:**
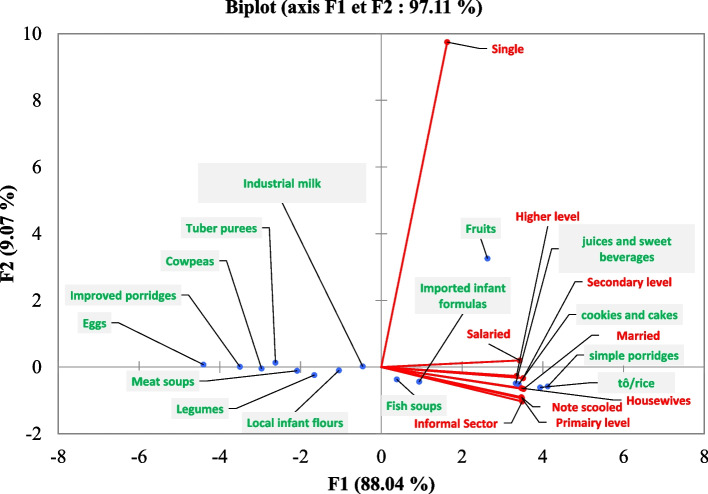
Principal Component Analysis of child food consumption according to the mother's social class

Principal component analysis of the different foods children consume shows various correlations according to the mother’s social status (Fig. 2). Consumption of imported infant formula, fish soups, fruits, juices and sweet beverages, cookies and cakes, simple porridges, and tô/rice positively correlated with the mother's marital status, education level, and occupation. The results also showed a strong association between the consumption of cookies, cakes, simple porridges, sweet juices and drinks, tô/rice with the status of married women, housewives, secondary education level women and women with higher education level. On the other hand, the children of women with no schooling level, women with a primary education level and women in the informal sector are much more associated with the consumption of imported infant flours and fish soups, although the correlation is slightly weak. Single women were not strongly related to the consumption of any food. In general, the consumption of eggs, improved porridges, cowpeas, tubers purees, meat soups, vegetables, local infant flours and industrial milk did not show a positive correlation and were not associated with any social status of the mother.

### Frequency of meal consumption by children

Of the total number of children, the frequency of meal consumption results showed that 33.98% of the children had a frequency of three meals per day, while 5.99% of children had a frequency of one meal per day (Table 3). As for breastfed children, children aged 9 to 23 months presented the highest meal frequencies compared to those aged 6 to 8 months. Regarding the minimum meal frequency of all children, 86.41% received the minimum meal frequency per day. Specifically, 86.10% of breastfed children received the minimum frequency, including 34.24% of children aged 6 to 8 months and 51.86% of children aged 9 to 23 months. As for the non-breastfed children, 92.86% had received the minimum frequency of four meals per day.

**Table 3:** Frequency of meals consumed according to the breastfeeding mode

Number of meals	Breastfed children (*N* = 590)		Non-breastfed (*N* = 28)	Totals (N)%	*P*-Value
	**6–8 months**	**9–23 months**	**6–23 months**		
One meal	(21) 3.56	(16) 2.71	(0) 0.00	(37) 5.99	0.01
Two meals	(65) 11.02	(45) 7.63	(0) 0.00	(110) 17.80	
Three meals	(78) 13.22	(130) 22.03	(2) 7.14	(210) 33.98	
Four meals	(23) 3.89	(71) 12.03	(7) 25	(101) 16.34	
Five meals	(28) 4.75	(76) 12.88	(12) 42.86	(116) 18.77	
Six meals	(8) 1.36	(29) 4.92	(7) 25	(44) 7.12	
Children receiving the minimum frequency of meals^a^	(202) 34.24	(306) 51.86	(26) 92.86	(534) 86.41	

*Legend*: *N* Number, *%* Frequency

^a^For breastfed children, the daily minimum frequency is two meals for children aged 6 to 8 months and three meals for children aged 9 to 23 months. As for non-breastfed children, the daily minimum frequency is four meals [21]

### Children's reactions to local infant meal porridges and mothers' perceptions

**Fig. 3:**
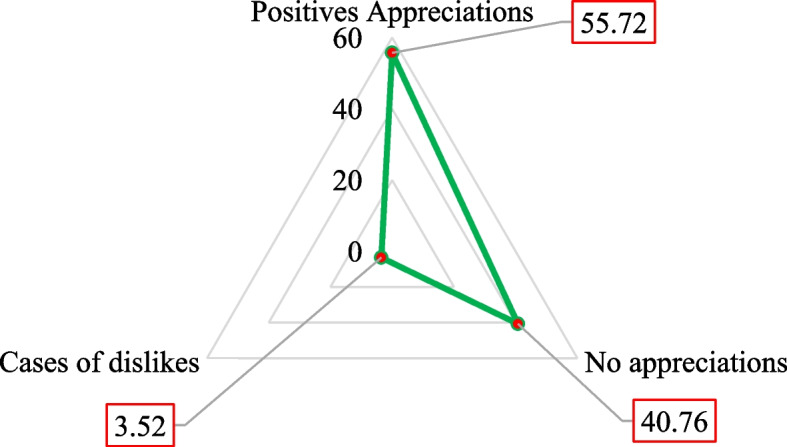
Children's reaction to local infant formula

**Fig. 4:**
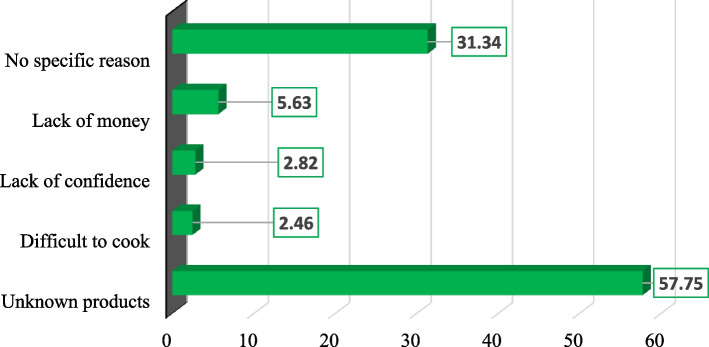
Women's perception of local infant formula

The results presented in Fig. 3 show that most children who consumed the local infant formula had positively reacted to it against 40.76% of the non-positive reactions. The main finding was that the rate of positive appreciation was higher than the rate of disliking, although the difference was insignificant. Cases of disgust with local infant formula were evaluated at 3.52%. The respondents gave several reasons to explain the low consumption of local infant formula (Fig. 4). Most women interviewed mentioned the lack of information about the availability of local infant formula. In contrast, 5.63% said the lack of resources, 2.46% of the women had noted the difficulty of preparation, and 2.82% cited the lack of confidence. The remaining 31.34% of the women gave no reason for non-use.

## Discussion

The results on the frequency of children consuming different types of food showed high consumption of cereal-based foods such as simple porridges, tô/rice, imported infant porridges, local infant porridges and improved porridges. These results corroborate those of other studies, which indicated a predominance of cereal-based food consumption, with 80.45% consumption of tô in households in Burkina Faso [22] and 97% consumption of cereals by children in Ivory Coast [23]. These results clearly show that household consumption patterns in Burkina Faso, dominated by high cereal consumption [24], influence children's food consumption. The low level of consumption of tubers could be justified by the high cost of these seasonal products, which were out of reach during the survey period. Children heavily consumed cookies and cakes, confirming the findings of other authors who also reported high consumption of cookies in France and Burkina Faso [25, 26]. The high consumption of cookies could be related to their great diversity, texture, variety of tastes, ease of use and conservation. The results on the consumption of juices, sweet beverages and fruits in the present study showed a high-frequency, contrary to the results of [23], which revealed only 20% of this consumption in Ivory Coast. This high consumption of sweet juices could be related to children's preference for sweet products [24], their slightly acidic taste [27] and the high fruit availability during the survey period. In general, meat products (meat and fish) are consumed at a low level, similar to other studies that found 32.7% of meat consumption in northern Burkina Faso [24]. The low consumption of these foods has also been noted among rural communities in Ethiopia [28]. This low consumption of meat products would be linked to socio-cultural allegations, as some respondents stated that children's consumption of meat, eggs and cowpeas was forbidden in their communities. According to some authors, a diet low in meat products could result in micronutrient deficiency and lead to malnutrition [29]. To supplement the low consumption of meat products, an author also recommended the consumption of vegetables to ensure balance, according to their protein and mineral composition [30]. However, the present study showed low consumption of vegetables and dairy products, similar to other studies done in Ivory Coast [23, 31]. Cowpea consumption (17.31%) is lower than those of another similar study carried out in Burkina Faso, which indicated between 48.3 and 44.7% consumption [24]. In general, eggs are very little consumed (6.63%). A recent study in Ivory Coast also found 14% of egg consumption [23]. This low consumption is strongly linked to socio-cultural claims. Indeed, during the survey, some respondents mentioned that in their communities, it is widely believed that the consumption of eggs and meat could influence children's language learning and their behavior as adults. The studies of Khalid et al*.* (2017) also considered the cultural aspect, the allergies and the lack of means in the low consumption of eggs in Ethiopia [32]. However, contrary to these claims, some authors have reported that early exposure and repeated exposure of children to different flavors, smells, and tastes of family meals would be an important factor promoting the future acceptability of foods at older ages [33, 34]. It is also recognized that early consumption helps prevent specific food allergies [11].

Of all the types of food consumed, only the consumption of tô/rice, local infant flour, improved porridge and fruit was significantly associated with infant breastfeeding mode. This significant correlation is related to parental beliefs and preferences. For most respondents, some foods such as cookies, cakes, juices and sugary drinks, imported infant flours and fish were considered to have vitamins in quantity. Similarly, cookies and cakes were associated with health recommendations. As for porridge and tô, they were considered to ease digestion. The correlation between the consumption of porridges, fish soups and tô/rice with the status of married women and unemployed housewives is explained by the fact that they are generally responsible for preparing the family meal, which consists mainly of cereals-base food. In the case of cookies, cakes and sweet juices, the prejudice on the vitamin composition would be one of the main reasons for their high consumption by women with secondary education and high education level. The strong correlation between the consumption of sweet juices and drinks with the situation of low-income unemployed housewives has also been demonstrated by Dubois (2005), who found that this type of food consumption increases with the low income-of mother in Quebec [35]. The lack of a positive correlation between the consumption of certain foods and the mother's social status could be explained by social stereotypes regardless of social status. This situation is greatly accentuated by the strong presence of the extended family in the care of children, the availability and accessibility of certain foods (industrial milk and derivatives) and the consumption habits of parents [24, 32, 36].

The frequency of meal consumption results showed that 33.98% of the children surveyed consumed three meals daily. This result is lower than those of another similar study that indicated more than 40% of children with frequencies of three meals per day in Burkina Faso [37]. As for the finding on the minimum frequency of meals, the result was satisfactory, with 86.41% of children generally receiving the minimum daily meal frequency. However, this rate is lower than those obtained in a similar study in Ivory Coast, with 99.00% of children receiving the minimum frequency of meals [23]. On the other hand, it is higher than those of another study conducted in Dakar, Senegal, which presented 71.3% as the rate of minimum frequency of meals [38] and that of the national nutritional survey in Burkina Faso, which highlighted a rate of 61.1% for the Center Region [39]. The high rate of children who received the minimum frequency of meals in this present study compared to those of the national nutrition survey could be explained by the difference in the survey area. The results of the national nutrition survey in the Central Region that included the surrounding villages, which often have different practices and cultures than the cities, could be the cause of the low rate observed. Indeed, these practices may contribute to the lower rate in the region as a whole. When comparing consumption frequencies, children aged 9 to 23 months generally have a higher frequency of meals per day, which may be related to the increase in the child's nutritional needs with age, according to some authors [31, 40]. The results of this study noted that non-breastfed children (92.86%) were the most likely to receive the minimum meal frequency. This situation could be explained by the need to compensate for the absence of breastfeeding. Indeed, some respondents with non-breastfed children said that given the small amounts consumed by the child, requests for meals were quite frequent every day.

Regarding the consumption of local infant porridges, the results showed that overall, 55.72% of the children had positively reacted. This result is lower than that of another study carried out in the Gnagna province in Burkina Faso, which showed 66% of positive reactions [41], as well as those of Hervet et *al.* (2004), which showed positive appreciations ranging between 51.2 and 80% in Burkina Faso [42]. The cases of disgust mentioned by the respondents could be partly explained by the food neophobia developed by some children during their food learning [43]. Other reasons could be mentioned, such as differences in children's reactivity to different flavors [44] and the lack of meal variation over the day. Indeed, during the data collection, a great monotony in the children's meals, such as the consumption of the same meal several times on the same day, was noted. The primary reasons for not consuming the infant porridge were the lack of information on the availability of local infant flours, the lack of means, the preparation constraints and the lack of confidence. Another study by GRET cited the same reasons in Ouagadougou but reported that local infant formula was relatively accessible [41].

## Conclusion

The objective of this study was to describe the socio-cultural influences on feeding habits and food consumption frequencies of children aged 6 to 23 months in Ouagadougou. The results showed that simple porridges, tô, cookies and cakes, juices and sweet drinks were the most consumed. Cowpeas, improved porridges and eggs were the least consumed. The frequency of consumption was also high. Thus, more than one-third of the children had a frequency of three meals, while a small proportion of the children had a frequency of one meal per day. A large majority of children received the minimum frequency of meals per day. According to breastfeeding mode, the minimum frequency of meals was received by more non-breastfed children (92.86%) than breastfed children (86.10%). Regarding infant formula consumption, more than half of all the children who consumed infant formula had positively reacted. In contrast, only a tiny proportion of children were disgusted by the local infant formula. Several reasons were given by the respondents, including lack of information and, to a lesser extent, preparation constraints, lack of confidence and lack of resources. In general, we note a high consumption of family meals by young children. To further develop this study, an observational study would be necessary to confirm and consolidate the results obtained.

## Data Availability

The data used in this study are available from the corresponding author upon request.
